# Evaluating an e-mental health program (“deprexis”) as adjunctive treatment tool in psychotherapy for depression: design of a pragmatic randomized controlled trial

**DOI:** 10.1186/s12888-014-0285-9

**Published:** 2014-10-08

**Authors:** Tobias Krieger, Björn Meyer, Kerstin Sude, Antoine Urech, Andreas Maercker, Thomas Berger

**Affiliations:** Department of Clinical Psychology and Psychotherapy, University of Bern, Bern, Switzerland; GAIA AG, Hamburg, Germany; Department of Psychology, City University London, London, United Kingdom; Deutsche Psychotherapeuten-Vereinigung e.V., DPtV, Berlin, Germany; Department of Psychopathology and Clinical Intervention, University of Zurich, Zurich, Switzerland

**Keywords:** Depression, Internet-based treatment, Adjunctive treatment tool, Randomized controlled trial (RCT)

## Abstract

**Background:**

Major depressive disorder (MDD) places a significant disease burden on individuals as well as on societies. Several web-based interventions for MDD have shown to be effective in reducing depressive symptoms. However, it is not known whether web-based interventions, when used as adjunctive treatment tools to regular psychotherapy, have an additional effect compared to regular psychotherapy for depression.

**Methods/design:**

This study is a currently recruiting pragmatic randomized controlled trial (RCT) that compares regular psychotherapy *plus* a web-based depression program (“deprexis”) with a control condition exclusively receiving regular psychotherapy. Adults with a depressive disorder (N = 800) will be recruited in routine secondary care from therapists over the course of their initial sessions and will then be randomized within therapists to one of the two conditions. The primary outcome is depressive symptoms measured with the Beck Depression Inventory (BDI-II) at three months post randomization. Secondary outcomes include changes on various indicators such as anxiety, somatic symptoms and quality of life. All outcomes are again assessed at the secondary endpoint six months post randomization. In addition, the working alliance and feasibility/acceptability of the treatment condition will be explored.

**Discussion:**

This is the first randomized controlled trial to examine the feasibility/acceptability and the effectiveness of a combination of traditional face-to-face psychotherapy and web-based depression program compared to regular psychotherapeutic treatment in depressed outpatients in routine care.

**Trial registration:**

ISRCTN20165665.

## Background

Major depressive disorders (MDD) are among the leading causes of worldwide disability [1]. Several meta-analysis have shown that web-based self-help interventions can contribute to depressive symptom reduction and there is considerable support for the use of the Internet for delivering evidence-based psychotherapy for depression [2-7]. However, several studies suggest that web-based self-help interventions without support seem to result in more modest outcomes and considerably higher dropout rates than treatments including regular web-based support from therapists or technically qualified persons [6,8-10].

There is growing knowledge about the effects of web-based interventions. However, research on combining web-based technologies with traditional face-to-face therapy, i.e., blended treatments, is sparse. Importantly, the term *blended treatment* includes any possible combination of regular face-to-face treatments and web-based interventions. For instance, treatment components of a web-delivered intervention may be integrated intensively and used during the face-to-face treatment, or, as in the current study, may rather be used as an adjunctive intervention. Since research on blended treatments is scarce, it remains to a large extent an open question *if* and *how* web-based interventions can be combined meaningfully with traditional forms of psychotherapy and whether this may have an incremental effect [11].

So far, only a few studies have investigated combinations of regular face-to-face therapy and web-based adjunctive tools in mental disorders. For example, there are promising results regarding symptomatology and acceptability for (palmtop-) computer-assisted psychotherapies in different anxiety disorders [12-14]. Furthermore, in a study in which 77 individuals seeking treatment for substance dependence were randomly assigned to regular face-to-face psychotherapy or regular face-to-face psychotherapy with biweekly access to a computer-based training in cognitive-behavioral therapy (CBT), participants in the blended condition submitted significantly more drug-negative urine specimens and tended to have longer continuous periods of abstinence during treatment [15]. Moreover, a 6-month follow-up showed significantly better durability of effects of the combined treatment over regular treatment for both self-report and urinalysis data [16]. These results were replicated in a larger randomized clinical trial in a more homogeneous clinical population, i.e., cocaine-dependent individuals maintained on methadone [17].

Regarding MDD and combined treatments, research is very sparse. For example, Wright and colleagues conducted an open trial of a computer-assisted psychotherapeutic treatment in a sample consisting predominantly of patients suffering from depression. Results indicated a high rate of patient acceptance of this form of treatment, and scores of symptom severity as well as the mean score on a measure of cognitive therapy knowledge were significantly improved [18]. Subsequently, Wright and colleagues [19] conducted a RCT comparing a conventional face-to-face cognitive therapy (CT), a wait-list, and computer-assisted cognitive therapy with reduced therapist contact in patients suffering from a MDD. Results indicated that the combined condition was more effective than the wait-list condition and equally effective as conventional CT. These effects were maintained over a 6-month period. Furthermore, computer-assisted cognitive therapy showed more robust effects, relative to being wait-listed, than standard cognitive therapy in reducing measures of cognitive distortion and in improving knowledge about cognitive therapy.

Recently, Mansson et al. [20] conducted a proof-of-concept study on the effectiveness of a blended form of psychotherapy in a mixed anxious-depressed sample. The adjunctive web-based platform was to be used at home and in the clinical setting where treatment was provided. The main part of the platform consisted mainly of material that would have been presented on paper or verbally in a face-to-face session. Additionally, the platform comprised some basic components of CBT, such as scheduling sessions, keeping an agenda, and setting goals. Results indicated that this blended treatment had a positive effect across anxious and depressive symptom measures as well as on a quality of life measure in a mixed sample of 15 patients suffering from MDD and/or anxiety disorders. Additionally, patients as well as therapists rated the treatment mostly favorably. Positive patients’ feedback included, for example: memory support and learning, potential to gain an overview of the treatment process, positive implications for homework assignments, promotion of a sense of autonomy and responsibility.

The literature cited above indicates that combining traditional face-to-face psychotherapy and web-based tools may be a promising venue. A possible explanation for these results is highlighted by a recent review, which concludes that patients’ treatment adherence may be improved through new technologies [21]. Potential benefits of blending treatments could for example be a greater reduction of depressive symptoms or more cost-efficacious treatments [19]. However, there could also be some serious downsides of combining conventional face-to-face treatments and new technologies. For example, a recent randomized controlled study showed that appointment reminder via SMS did not lead to higher appointment attendance as expected and, more importantly, that more client dropped out in the reminder condition compared to the no reminder condition [22]. Finally, to our knowledge there is no study that investigates how the blending of treatments affects important process variables such as the therapeutic alliance.

### Objective and research questions

The main objective of the present study is to further investigate combined treatment approaches for MDD by evaluating an empirically validated web-based treatment (*deprexis;* [23-25]) as an adjunctive tool in regular psychotherapeutic treatment in comparison with traditional psychotherapy in a sample of depressed outpatients by means of a pragmatic RCT in routine care. Therefore, our main hypothesis is that the combined treatment (TAU plus Deprexis) is more effective than regular psychotherapeutic treatment (TAU) alone.

Additionally, during a 6-month period we intend to test on an exploratory basis how helpful patients as well as therapists rate the combined treatment and whether the combination is associated with increased efficiency (i.e., reduction of number of sessions) or with negative side effects (e.g., lower quality of the therapeutic alliance, increased drop-out rate).

## Methods/design

### Design

**Figure 1:**
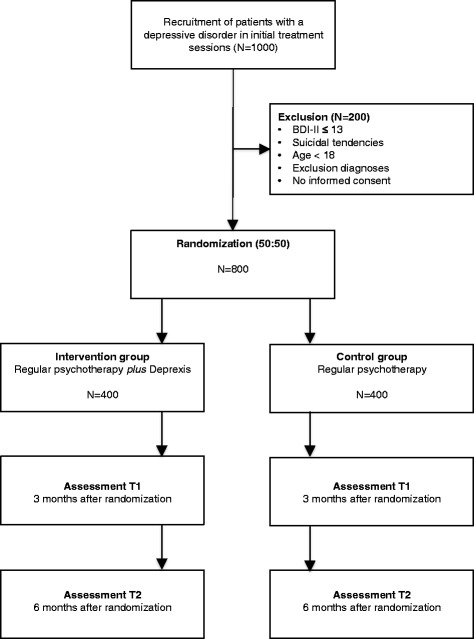
**Study design.**

A two-armed randomized pragmatic RCT is currently being conducted to compare regular face-to-face psychotherapeutic treatment with a combined treatment, i.e., face-to-face psychotherapeutic treatment *plus* a web-based adjunctive treatment tool (*deprexis*). Measurements in both conditions are assessed at baseline (T0), after six weeks (process measures), after three months (T1), and after six months follow-up (after randomization) (T2). The study design is shown in Figure 1.

### Ethics

The study is being conducted in compliance with the Declaration of Helsinki and has been approved by the Ethics Committee of the German Psychological Association (DGPs, TB072013; 16/08/2013) and the Hamburg Chamber of Psychotherapists (18/12/2013). Participants are informed that they can withdraw at any time without having to disclose reasons. Informed consent is obtained online prior to the baseline assessment. All participants receive oral and written information from their therapist about the aim of the study, benefits and risks of participation and the study procedure.

### Inclusion and exclusion criteria

We include adults (a) with age 18 and above, who (b) suffer from a MDD according to the International Classification of Diseases (ICD-10; i.e., F32.-depressive episode, F33.- Recurrent Depressive Disorder, F34.- Persistent Affective Disorder, F38.- Other Affective Disorder, F39 Unspecified Affective Disorder), (c) have a Beck Depression Inventory (BDI-II) sum score over 13, (d) have sufficient knowledge of the German language, (e) have Internet access and sufficient knowledge to use it (based on self-report), and (f) are willing to give informed consent. All participants are recruited in Germany.

We will exclude subjects who (a) have a known psychotic or bipolar disorder, (b) suffer from a chronic depression with onset in childhood (based on clinical judgment), and (c) show a notable suicidal risk (based on clinical judgment of the therapists).

### Recruitment

Forty therapists, all members of the Deutsche PsychotherapeutenVereinigung (DPtV), working in secondary care will be recruited. All therapists are informed about the study by the DPtV and all interested members are invited to participate in the study. Participating therapists inform their self-referred patients about the study and ask them if there are willing to take part in the present study. Potential study participants (patients) are informed about the possibility of taking part in the present study in the initial therapy session by their therapist. Interested patients receive an information sheet and a written informed consent form by the therapists and are invited to ask questions. After returning the completed informed consent form individually to the therapists, participants are asked to complete online screening questionnaires at home to assess whether they fulfill the inclusion criterion of BDI-II-Score > 13.

### Randomization

Participants who return the informed consent and meet all of the inclusion and none of the exclusion criteria will be randomly allocated to the study conditions (50:50). Randomization takes place at an individual level within therapists. Thus, therapists provide both treatments, allowing us to control for therapist effects. The allocation list will be produced using an automated computer-generated random numbers table placed in a secured web-based database and will be concealed to the investigators and the therapists (www.random.org). After randomization, the participant and his or her therapist will be informed about the condition by email.

### Blinding

In the present study, there is no blinding implemented, consistent with recommendations for the conduct of pragmatic RCTs [26]. All participants will be informed about the aims and the methodology of the study. They can ask questions regarding the study and withdraw their informed consent at any time. The focus of the present study is on external validity and generalizability of the results to routine clinical practice.

### Description of the interventions

#### Intervention group: Psychotherapy plus web-based online platform (deprexis)

Participants in the intervention group receive access to the *deprexis* platform. Trained, certified psychotherapists introduce the patients to the use of the program. There are no costs for patients or therapists for the program use during the study.

*Deprexis* provides psychoeducational information and exercises that are mainly based on cognitive-behavioral psychotherapy and aim at decreasing depressive symptoms. *Deprexis* includes ten modules plus one summary module: (1) psychoeducation, (2) behavioral activation, (3) cognitive restructuring, (4) mindfulness and acceptance, (5) interpersonal skills, (6) relaxation, (7) physical activity and lifestyle modification (e.g., exercise and nutrition), (8) problem-focused approaches, (9) expressive writing and schema-focused contents, (10) positive psychology as well as emotion-focused-interventions. A more comprehensive description of the program can be found in Meyer and colleagues [23].

Communication between the participant and *deprexis* takes place over simulated online-dialogues in which the user reacts to the content by clicking on multiple-choice answers, e.g., in order to express doubts, to confirm particular contents, or to request further information. Text units are illustrated with drawings, pictures and audio clips. The modules can be accessed repeatedly during the intervention interval. Depending on the reading speed of the participants the completion of a module takes between 10 and 60 minutes.

Furthermore, *deprexis* includes short questionnaires for the assessment of current mood, which offers the possibility to visually inspect the mood trajectory over the course of therapy. Additionally, the program provides the user with printable summaries and work sheets. Optionally, the user receives daily short text messages to remind him or her to use the program and reiterate program content.

Therapists can support participants with the program use following clinical judgment and discuss progress with them during face-to-face sessions. For this purpose, therapists will be introduced to the content of the program and the “therapist cockpit” in a three-hour workshop. In the therapist cockpit, therapists can track participants’ program use and symptomatic progress. In the workshops, therapists are informed that the program can be regarded as an adjunctive tool that need not influence the content of their face-to-face sessions. For continuous support, participating therapists can contact the program developer and owner (GAIA AG, Hamburg) by phone or email as required.

### Control group: Psychotherapy without Deprexis

Patients in the control group receive regular psychotherapy, i.e., usually weekly one-hour sessions, according to the clinical judgment of the therapists. The behavior of the therapists and the number of therapy sessions are not deliberately influenced by the study. In case of interest, participants in the control condition will receive access to *deprexis* after completion of the study.

### Therapists

All therapists are members of the DPtV. With over 10,000 psychotherapists the DPtV is the largest advocacy for approved psychological psychotherapists and child and adolescent psychotherapists in Germany. Most participating psychotherapists in the study identify themselves as being eclectic with a focus on cognitive-behavioral therapy, but also psycho-dynamically oriented therapists take part in the study.

### Sample size calculation

Regarding differences between the two active treatment conditions, we want to be able to detect standardized between-groups effects sizes (Cohen’s *d*) of 0.2. Smaller effect sizes are considered to be negligible from a clinical point of view [27]. At an α error level of .05 (two-tailed), a statistical power (1-Beta) of .80, and a correlation between pre- and post-measurements found in our earlier studies, we need to include 800 participants. This sample size calculation is conservative and is based on a current meta-analysis in which a mean effect of *d* = .28 for online depression programs was found [5]. Since in the current study participants in both conditions will receive psychotherapy, a significant reduction of depressive symptoms can also be expected in the control condition. Therefore, a relatively small effect size between conditions, i.e., depression level after three months between *deprexis* plus psychotherapy vs. psychotherapy is expected.

### Primary and secondary outcomes

Primary outcome is depressive symptoms assessed with the Beck Depression Inventory, second edition (BDI-II). In secondary analyses, we will explore the effects on quality of life, anxiety, somatic symptomatology, health-related quality of life, psychological aspects of the treatment process, and psychological empowerment. All measurements are assessed online. In order to promote completion when assessments are missing, participants will be automatically reminded twice to complete the assessments. For an overview of all outcome measures, see Table 1.

**Table 1:** **Outcome measures**

	**Patients**	**Therapists**
T0 (Baseline)	• Demographic Variables Questionnaire	• Questions regarding the theoretical orientation
	• Depression: BDI-II	ICD-10 diagnoses of the patient
	• Suicidal tendencies: SBQ-R	
	• Web Screening Questionnaire (WSQ)	
	• Anxiety: GAD-7	
	• Somatic symptoms: PHQ-15	
	• Health-related quality of life: SF-12	
	• Questionnaire for the evaluation of psychotherapy courses (FEP-2)	
	• Empowerment: Psychological Empowerment Scale	
6 weeks	Working Alliance Inventory (patient - perspective)	Working Alliance Inventory (therapist - perspective)
12 weeks	Working Alliance Inventory (patient - perspective)	Working Alliance Inventory (therapist - perspective)
T1 (Primary endpoint) 3 months	As baseline, plus:	Assessment of acceptability of treatment in psychotherapy
	• Subjective rating of the treatment course	
	In intervention group: Subjective rating of treatment	
T2 (Secondary endpoint) 6 months	As Baseline, plus:	Assessment of acceptability of treatment in psychotherapy
	• Subjective rating of the treatment course	
	In intervention group: Subjective rating of treatment	

### Outcome measures

#### Depressive symptomatology

The German version of the BDI-II [28] will be the primary outcome measure. The BDI-II is a widely used self-report measure for the assessment of depression severity with good psychometric properties and a completion time of 5-10 minutes. Analogous to a previous study [25] on the efficacy of online depression treatment, we only include patients with a score greater than 13. Scores from 0-13 indicate no or minimal depressive symptoms, scores from 14-19 indicate mild depression, from 20-28 moderate depression, and from 29-63 severe depression.

### Web Screening Questionnaire (WSQ)

The WSQ is a 15-item self-report instrument screening for frequent mental disorders [29]. Evidence indicating adequate diagnostic validity has been reported for social phobia, panic disorder with agoraphobia, agoraphobia without panic disorder, obsessive-compulsive disorder and alcohol abuse/dependence (sensitivity .72-1.00; specificity .63-.80) [29]. Somewhat more modest psychometric properties have been reported for major depressive disorder, generalized anxiety disorder, posttraumatic stress disorder, specific phobia and panic disorder without agoraphobia (sensitivity: .80-.93; specificity: .44-.51) [29].

### Suicidal tendencies

Current suicidal tendencies will be assessed by means of the Suicidal Behaviours Questionnaire-Revised (SBQ-R) [30]. This self-report measure consists of four questions regarding lifetime suicidal tendencies, the frequency of suicidal thoughts during the last 12 months, the course of suicidal intentions, and the probability of suicidal behavior in the future. The SBQ-R has shown high internal consistency. Furthermore, it has shown clinical utility in that scores on the instrument differentiated between subgroups of suicidal and nonsuicidal youth and adults [30].

### Anxiety

Symptoms of anxiety are assessed with the German version of the 7-item Generalized Anxiety Disorder Scale [31] in order to identify patients with a generalized anxiety disorder and to assess anxiety symptoms. The GAD-7 consists of seven items that correspond to the diagnostic criteria for GAD from the DSM-IV. The GAD-7 has good internal consistency and good convergent validity with other anxiety scales [32].

### Somatic symptomatology

To assess the level of somatic symptoms, patients complete the somatic symptom module of the Patient Health Questionnaire (PHQ), the PHQ-15 [33]. The items include the most prevalent somatic symptom complaints reported in primary care. Each symptom is scored from 0 (“not bothered at all”) to 2 (“bothered a lot”). The PHQ-15 has shown to be equal or superior to other brief measures for assessing somatic symptoms and screening for somatoform disorders [32].

### Quality of life

To assess quality of life, patients complete the Short-Form Health Survey-12 (SF-12). The SF-12 is based upon the Short-Form Health Survey (SF-36). Its two subscales measure physical and mental aspects of health-related quality of life. It captures general health as well as pain, disabilities in daily life and mental problems. The SF-12 asks for the presence and severity of 12 items over the course of the last four weeks. The re-test reliability is good and it is roughly equivalent to the long form [34].

### Psychological aspects of the treatment process

The Questionnaire for the evaluation of psychotherapeutic progress (FEP-2) is a measure of therapeutic progress and can be used for both change and outcome assessment [35]. Forty items measure the dimensions well-being, symptoms, interpersonal relationships, and incongruence with respect to approach and avoidance goals. The instrument has shown to be change sensitive as well as reliable and it is available in the public domain. It represents the phase model of therapeutic change as well as interpersonal and integrative models of psychotherapy [36].

### Psychological empowerment scale

Empowerment is a psychological motivational construct, which includes for domains: competence, meaning, self-determination, and impact. Psychological empowerment will be assessed with an adapted version of the scale by Spreitzer [37,38].

### Process measure

After six and 12 weeks the cooperation between the patient and the therapist will be assessed with the German version of the Working Alliance Inventory – Short (WAI-S; adapted version) [39]. The WAI-short (WAI-S) is a 12-item self-report measure of Working Alliance. Each item is rated on a 7-point scale, with higher scores indicating higher alliance. The WAI-S is scored into three subscales measuring Task, Goal and Bond. The Task and Goal scales are intended to measure agreement between the client and therapist with regard to Tasks and Goals of the treatment. The Bond subscale aims to measure the empathic bond between the client and the therapist [40].

### Other measurements

Patients are self-reporting a number of demographic variables including sex, marital status, level of education. Additionally, we assess the patients’ medication status.

### Therapist measurements

We collect information from the therapists such as the diagnoses based on clinical judgment, number of therapy sessions, and self-reported usefulness and implementation of *deprexis* in mental care. Therapists’ variables such as age, gender, self-identified theoretical orientation, and years of experience are collected from therapists through a questionnaire.

### Statistical analyses

Primary and secondary outcomes will be conducted on the intention-to-treat (ITT) sample. A linear mixed-model repeated measures ANOVA with time as a within groups factor and treatment condition as a between-groups factor will be used for the main research question. Mixed-model repeated measures ANOVA uses all available data of each subject and does not involve the substitution of missing values [41]. With regard to the process variable, i.e., the quality of the working alliance, we will conduct Pearson correlations of the WAI with residualized change scores on the primary and secondary outcomes.

## Discussion

Major depression is a prevalent and debilitating disorder. Even though there is ample evidence for the efficiency and effectiveness of (guided) Internet-based interventions as well as for conventional psychotherapeutic treatments for depression, there are various strengths and limitations for both forms of treatments [11].

In this study, we will examine the effectiveness of psychotherapy plus a web-based online depression program (*deprexis*) versus traditional psychotherapy in routine care. We expect that depression levels of participants in the intervention group will be significantly lower after three months and after six months compared to the control group. Additionally, we will gain valuable experiences in combining an online psychological intervention with routine psychotherapeutic care.

A major strength of this study is that to the best of our knowledge, it will be the first study that tests whether an adjunctive web-based treatment tool in psychotherapy for depression can be successfully integrated in routine care and whether the combination will show additional effects compared to a regular psychotherapy for depression.

There are important limitations in the present study that should be mentioned: First, we adopted a crossed therapist design, i.e., a given therapist delivers the control as well as the experimental condition. This design is associated with potential drawbacks [42] but was necessary to choose in order to be able to run the study in routine care. In order to adjust for the potential downsides of this design, in the analyses we will control for therapist allegiance. Second, we will not have standardized assessments of depression diagnosis but rely solely on clinical diagnoses of experienced, licensed psychotherapists plus an online self-report diagnostic assessment. Third, in the present study, we will use *deprexis* as the only adjunctive web-based intervention tool. Therefore, generalizability of the results to other web-based interventions will be restricted.

In addition to the main analysis described in the methods section, our study will be able to address a number of other important research questions, some of which have not been addressed before. For example, we will be able to assess whether an adjunctive web-based treatment affects working alliance scores. Furthermore, we will assess attitudes towards blended psychological interventions from the patients’ as well as the therapists’ perspective.

If leading to a larger improvement, using effective web-based programs such as deprexis as an adjunctive treatment tool could be an interesting option to consider in future mental health care. Additionally, this study will also give first impressions whether blending face-to-face psychotherapy with a web-based adjunct will have negative side effects, i.e., symptom deterioration or decrease of the patient rated quality of the therapeutic alliance. In general, results of this study will provide an informative basis whether a combination of traditional face-to-face psychotherapy and web-based depression program is feasible and whether it is more effective compared to regular psychotherapeutic treatment in depressed outpatients in routine care.

### Trial status

Currently recruiting (N_current_ = 28 as of 800).
